# A Revised Abaqus^®^ Procedure for Fracture Path Simulation Based on the Material Effort Criterion

**DOI:** 10.3390/ma17163930

**Published:** 2024-08-07

**Authors:** Jakub Gontarz, Jerzy Podgórski

**Affiliations:** Department of Civil Engineering and Architecture, Lublin University of Technology, 20-618 Lublin, Poland; j.podgorski@pollub.pl

**Keywords:** fracture mechanics, fracture criteria, Abaqus^®^ user subroutine, X-FEM

## Abstract

This paper presents the results of computer simulations of fracture in three laboratory tests: the three-point bending of a notched beam cut from sandstone, the pull-out test of a self-undercutting anchor fixed in sandstone, and the pull-out test of a bar embedded in concrete. Five material failure criteria were used: Rankine, Coulomb–Mohr, Drucker–Prager, Ottosen–Podgórski, and Hoek–Brown. These criteria were implemented in the Abaqus^®^ FEA system to work with the crack propagation modeling method—extended finite element method (X-FEM). All criteria yielded similar force–displacement relationships and similar crack path shapes. The improved procedure gives significantly better, close-to-real crack propagation paths than can be obtained using the standard subroutines built into the Abaqus^®^ system.

## 1. Introduction

Numerical fracture simulation is a convenient and frequently used method for determining the critical states of materials and structures. However, it requires specialized software that, in addition to determining the critical load values, can determine the shape and range of the propagating crack. This is especially important when studying brittle or quasi-brittle materials, where the singularities of the stress field near the crack tip are the cause of large errors in determining the parameters of the propagation process. Such materials can include, for example, concrete and hard rocks, such as sandstone, granite, and dolomite, for which the fracture process is characterized by great dynamics. These are materials often tested in pull-out tests to determine the safety and load-bearing capacity of anchors fixed in walls or the size of a chipped rock fragment and the force required to break it off [1,2,3,4,5]. This is one of the possible rock removal methods tested for mine rescue [6]. One of the software tools that can be used for these simulations is the advanced and popular Simulia Abaqus^®^ system [7], which offers techniques to perform this type of study. Using this system in a ‘standard’ way, however, comes with some limitations that, under certain conditions, produce simulation results that are not very accurate in relation to predicting the direction of crack propagation. These limitations can be overcome thanks to the openness of the Abaqus^®^ system, which enables the implementation of independently developed subroutines that control the calculations.

This paper continues the authors’ work [8] on their own method for simulating crack propagation in the Abaqus^®^ FEA system. This method uses selected material failure criteria and determines the crack initiation and propagation angle. It cooperates with the X-FEM method to simulate the crack propagation. The authors’ method was implemented in Fortran as an Abaqus^®^ User Subroutine [7]. The main goal of this research is to compare several material failure criteria that were implemented in the Abaqus^®^ system by the authors and to check if it is possible to easily implement any criterion to work with the Abaqus^®^ system.

The X-FEM method is one of the most popular fracture simulation methods used in FEA systems [9]. The crack is implemented by finite element separation using an enrichment function added to the element shape function, which allows the simulation of displacement discontinuities in the element area. Typically, programs support a single crack jumping from edge to edge of an element during simulation in two-dimensional models, but solutions to three-dimensional problems can be found in the literature [10] and even provide crack branching [11].

Simulia’s Abaqus^®^ FEA system enables complex simulations of nonlinear processes occurring in brittle and quasi-brittle materials during cracking. This program allows the use of many different crack simulation methods, including the X-FEM method in combination with the Cohesive Zone Method (CZM), which makes it possible to achieve a high degree of independence of the simulation results obtained from the FEM mesh density. The (CZM) technique of avoiding the singularity of the stress field in the vicinity of the crack tip, proposed by Barenblatt [12], is still frequently used in fracture simulation analyses of brittle materials and continues to be improved and developed by today’s researchers [13,14].

**Figure 1:**
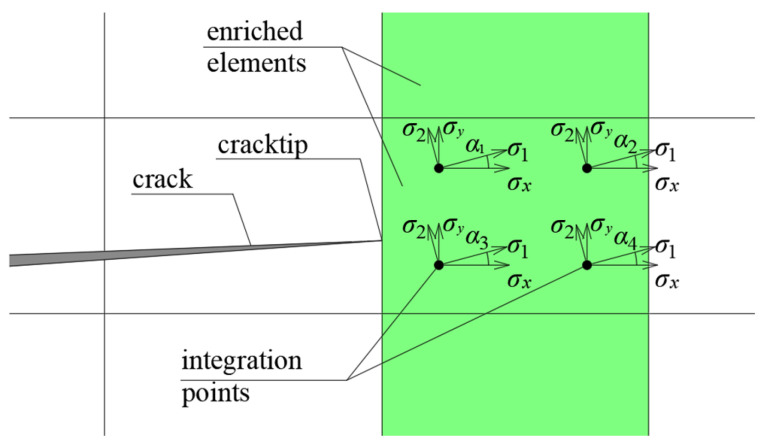
Rotation of stress tensors to principal stresses at integration points in an enriched finite element.

The Abaqus^®^ system allows the selection of several default criteria for determining the crack initiation and propagation angle. The most typical criterion for applications in homogeneous materials, in which the crack can run in any direction, is the MAXPS criterion, i.e., the criterion of maximum principal stresses. There are several different criteria that cooperate with X-FEM formulation in Abaqus^®^, such as MAXS (maximum nominal stress) or QUADS (quadratic nominal stress), but they are not fit for analyses of brittle materials—by selecting these criteria, the slot can only be routed in directions along the axes of the local finite elements. For the purposes of this paper, the MAXPS criterion will be called the default or built-in criterion. It works in the following way: the program reads the stresses at the integration points of the element to which the crack tip belongs, and the fracture propagation angle is equal to the average of the rotation angles of the stress tensors to the principal stresses at four integration points. Fracture occurs when the average maximum principal stress exceeds the specified tensile strength. A diagram of how this method works is shown in Figure 1.

The X-FEM technique implemented in Abaqus^®^ is quite immune to the density of the finite element mesh. Abaqus^®^ does not lead the stresses to infinity at the crack tip—the maximum value is close to the tensile strength; therefore, regardless of the size of the finite elements, the destructive forces (i.e., peak forces) in the model are very close to each other, which was proven in several previous author’s works. However, the difference in mesh size may lead to differences in the crack line, as Abaqus^®^ always leads the crack from edge to edge of the element. This may cause a crack in a model with a very small number of finite elements that are not as smooth as in a model with a dense mesh. However, the simulations analyzed below have enough finite elements to consider the resulting cracks smooth enough.

## 2. Materials and Methods

### 2.1. Own Method for Selecting the Direction of Fracture Propagation

**Figure 2:**
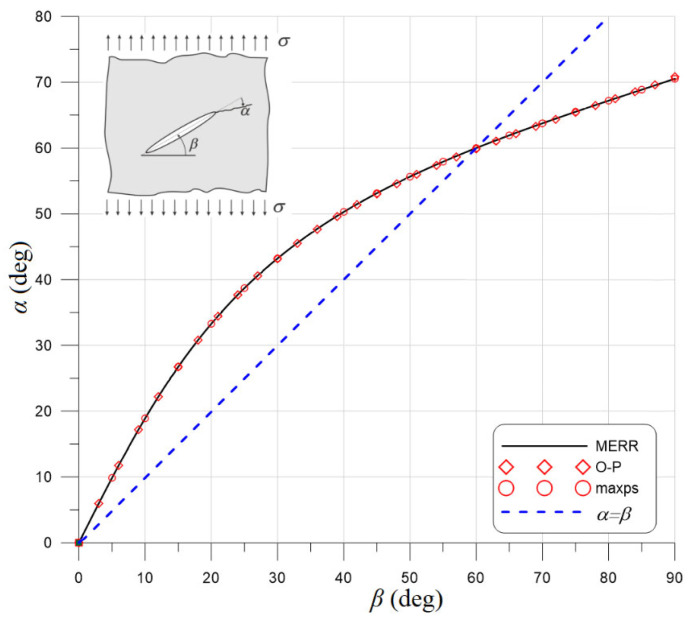
Dependence between crack propagation angle α and initial crack angle *β* for a Griffith’s crack for programmed criteria and the maximum energy release rate (MERR) criterion. O-P: minimum gradient of the effort function for the Ottosen–Podgórski failure criterion, maxps: maximum principal stress, *α* = *β*: direction normal to applied stress.

The method for predicting the direction of crack propagation was based on the authors’ proposal using the direction of the material effort field gradient [15]. It provides results almost identical (Figure 2) to the classical maximum energy release rate method [16] and is much more convenient and easier to apply for complex analyses [8].

This method for selecting the direction of crack propagation was implemented in the Abaqus^®^ system using the Abaqus^®^ User Subroutine and written in Fortran programming language. The most important subroutine used in the simulations is the UDMGINI (User Damage Initiation), which Abaqus^®^ runs on every iteration of the calculations (even several thousand times during the simulation). The input data of the subroutine are the stresses and coordinates at the integration points of the enriched elements and other available data that can be imported from other subroutines. The output data is only the value of the material effort and the crack propagation angle. If the subroutine determines the material effort to be greater than 1, Abaqus^®^ leads the crack to the next finite element. The authors also wrote two other auxiliary subroutines: UVARM (user subroutine to generate element output) and URDFIL (user subroutine to read the result file).

**Figure 3:**
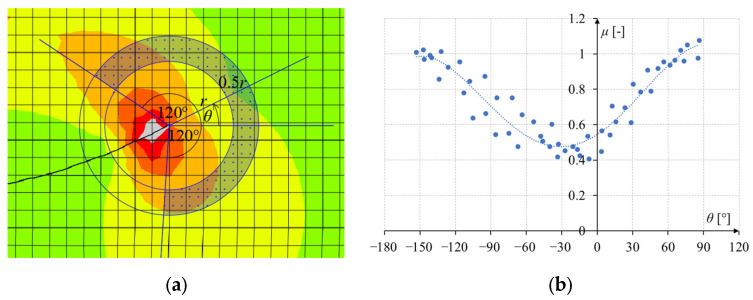
Method of determining the direction of crack propagation: (**a**) example of selecting integration points taken into account in determining the material effort plot; (**b**) example of the relationship between material effort and the angle around the crack tip.

The subroutine codes and the operation of the authors’ method for determining the direction of crack propagation are described in the authors’ previous publication [8]. The idea of how the algorithm works is simple. For each load increment, the program selects several dozen integration points around the crack tip, reads the stresses at these points, and then uses the stresses to calculate the material effort, which depends on the selected material failure criterion. The effort value is then interpolated to points lying equidistant from the crack tip. Figure 3b shows an example of a graph of the relationship between the values of the material effort at the integration points and the angle θ between the horizontal axis and the line connecting the crack tip with each integration point. The interpolation polynomial is then fitted to points with these coordinates, and then the subroutine finds its local minimum. The angle for which the local minimum is obtained is the angle of the next step of crack propagation. Additionally, the crack propagates when the material effort at the integration point closest to the crack tip is greater than 1. This is a better method than the built-in method, where the crack is determined by the average from the four integration points.

The method of selecting integration points taken into account for the polynomial fitting stage was developed by trial and error. There should be enough points to fit the polynomial as accurately as possible. These points should not be too close to the crack tip, as there are quite large disturbances. However, they should be close enough due to the fracture being determined by the stresses in a small area around the crack tip. It was decided that the algorithm would select the radius *r* so that there are 50 to 100 integration points at a distance from *r* to 1.5 *r* from the crack tip. Too large a number would also lead to an unnecessary increase in the duration of the simulation. Moreover, only integration points that lay a maximum of 120° to the left and right of the angle of the existing crack are taken into account in the calculations. This angle could not be too large to reject the stresses at points close to the already existing crack. It also could not be too small, or it would not provide enough integration points for calculations. An illustration of the method of selecting integration points is shown in Figure 3a. Also, by trial and error, it was decided that the polynomial fitting to the points would be of the fourth degree. Too low a degree of the polynomial caused the function to fit the points inaccurately in some cases.

**Figure 4:**
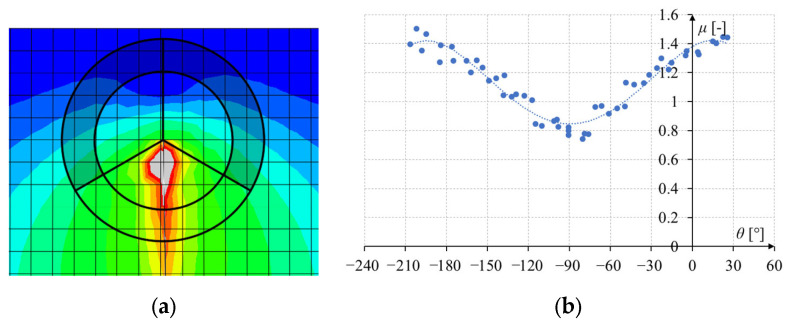
Example of the value of material effort around the crack tip in the case of a crack perpendicular to the edge of the model: (**a**) area of collecting the integration points; (**b**) relationship between material effort and the angle around the crack tip.

**Figure 5:**
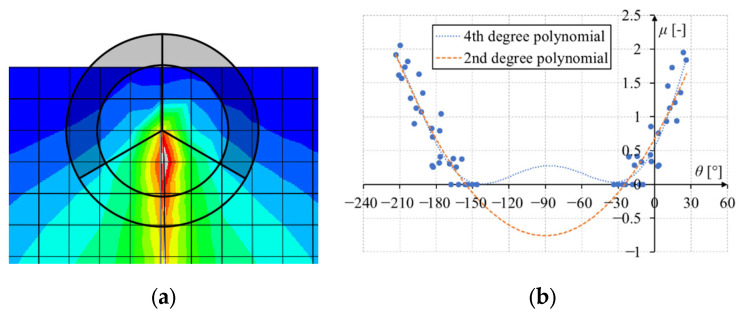
Example of the value of material effort around the crack tip in the case of a crack perpendicular to the edge of the model and with the crack tip close to the edge: (**a**) area of collecting the integration points; (**b**) relationship between material effort and the angle around the crack tip.

The selection of integration points was made as best as possible, but some problems still arose. Figure 4a shows a crack running perpendicular to the edge of the model. The area of collecting integration points overlapped the model, so the fit of the 4th-degree polynomial to the material effort values was not problematic, which is also visible in Figure 4b. Unfortunately, when the crack approached the edge, the area of collecting integration points partially went outside the model (Figure 5a). For this reason, for a large range of the angle *θ*, there were no data of stresses at the integration points, as can be seen in Figure 5b. In this case, fitting the 4th-degree polynomial was incorrect, as can be seen in the figure. The local minimum suddenly jumped to the wrong angle, causing the crack in the simulations to turn incorrectly to the left or right. This problem was solved in such a way that when the algorithm encounters a situation where a large part of the area taken into account for collecting integration points goes beyond the model, the polynomial is changed to the 2nd degree. Figure 5b shows that the local minimum for the 2nd-degree polynomial was correct for an angle of −90°, and the crack still went vertically.

**Figure 6:**
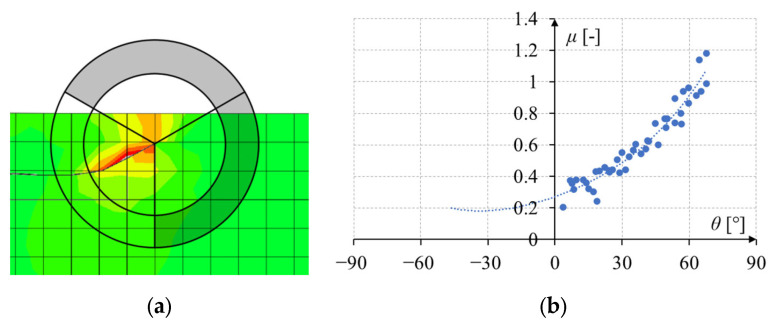
Example of the value of material effort around the crack tip in the case of a crack at a high angle to the edge of the model and with a crack tip close to the edge: (**a**) area of collecting the integration points; (**b**) relationship between material effort and the angle around the crack tip.

There is also another problem that seems to have no simple solution. When the crack approaches the edge of the model at a sharp angle, the area of collecting integration points sticks out beyond the model completely on the left side of the crack, as shown in Figure 6a. Theoretically, the crack should continue at the same angle *θ* as before (about −30°), but, as can be seen in Figure 6b, this angle is outside the range of the integration points taken into account. This leads to the fact that fitting any polynomial is highly inaccurate. In such a case, most often, the simulation from this moment gives a very unlikely crack path. Fortunately, this problem is not so significant, as this phenomenon occurs very close to the edge of the model, i.e., at the very end of the simulation.

### 2.2. Rankine Criterion

Five stress-based material failure criteria were programmed [17,18,19]. The first criterion was the Rankine criterion, which was programmed by the authors earlier and has already been used for the authors’ previous research.

**Figure 7:**
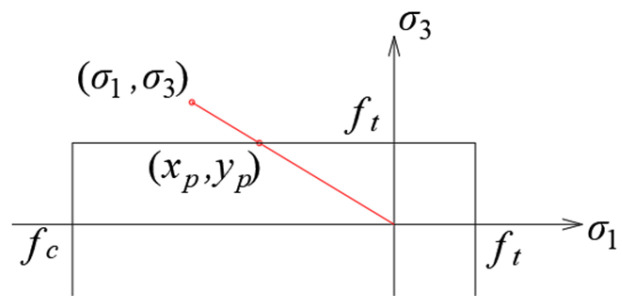
Envelope of the Rankine criterion in the plane of principal stresses.

The criterion envelope in the 2D stress state in the principal stress plane is shown in Figure 7, where *σ*_1_ and *σ*_3_ are the maximum and minimum principal stresses, and *f_t_* and *f_c_* are the tensile and compressive strength of the material, respectively. For the purposes of the analyses, tensile stresses were assumed to be positive, and compressive stresses were assumed to be negative. In theory, material fracture is influenced by both tensile and compressive strength. However, it was noticed in the simulations that it is not possible for the compressive stresses to exceed the compressive strength before the tensile stresses exceed the tensile strength. Therefore, it was decided to leave the value of material effort *μ* in the simplest possible form:

(1) $$\mu =\frac{\sigma_{1}}{f_{t}},$$

It should be remembered that cracking occurs when *μ* is greater than 1, that is, when the maximum principal stress point lies outside the criterion envelope (*σ*_1_ > *f_t_*). It is also worth noting that the criterion in this form does not require the compressive strength value.

### 2.3. Coulomb–Mohr Criterion

**Figure 8:**
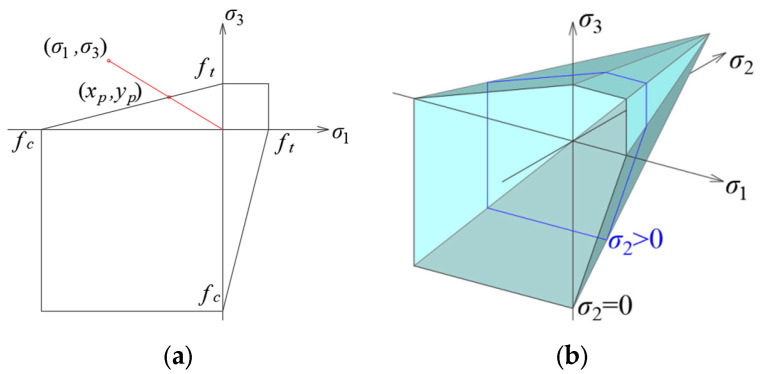
Envelope of the Coulomb–Mohr criterion in the plane of principal stresses: (**a**) in a two-dimensional stress state; (**b**) in a three-dimensional stress state.

In the case of the Coulomb–Mohr criterion in the compression–tension quadrant, the envelope in the 2D stress state in the plane of principal stresses takes the shape of a linear function (Figure 8a). The material effort is the ratio of the resultant stress values to the proportional stresses located on the criterion envelope:

(2) $$\mu =\frac{\sqrt{\sigma_{1}^{2}+\sigma_{3}^{2}}}{\sqrt{x_{p}^{2}+y_{p}^{2}}},$$

The intersection of the envelope with the line connecting the center of the coordinate system with the stress data point can be determined using simple formulas:

(3) $$x_{p}=-\frac{\eta }{1+\kappa \cdot \eta }\cdot f_{t},\;\;\;\;\;\;\;y_{p}=-\kappa x_{p},$$

where:

*η*—ratio of compressive strength to tensile strength *f_c_*/*f_t_*;

*κ*—ratio of maximum principal stresses to minimum *σ*_1_/*σ*_3_.

The purpose of the discussed method for predicting the direction of crack propagation is also the ability to determine the direction in tasks with a triaxial stress state (for example, axially symmetric tasks), i.e., when the middle principal stresses are different from 0. As can be seen in Figure 8b, a cross-section of the envelope with the *σ*_1_, *σ*_3_ plane will be different depending on the value of *σ*_2_ (an example cross-section for *σ*_2_ > 0 is marked with a blue line in this figure), so the method described in the above formulas will be incorrect. However, the material strength can be determined from the following formula:

(4) $$\mu =\frac{\eta +1}{2f_{c}}\cdot \max\left(\left|\sigma_{1}-\sigma_{2}\right|+K\left(\sigma_{1}+\sigma_{2}\right),\left|\sigma_{1}-\sigma_{3}\right|+K\left(\sigma_{1}+\sigma_{3}\right),\left|\sigma_{2}-\sigma_{3}\right|+K\left(\sigma_{2}+\sigma_{3}\right)\right),$$

where

(5) $$K=\frac{\eta -1}{\eta +1},$$

The advantage of the above formula is that it works for any stress configuration (compression–tension and tension–tension quadrant) and for a three-dimensional stress state. This formula, due to its relative ease of application to that described by Equation (2), was used in the algorithm implemented in the Abaqus^®^ system.

### 2.4. Drucker–Prager Criterion

**Figure 9:**
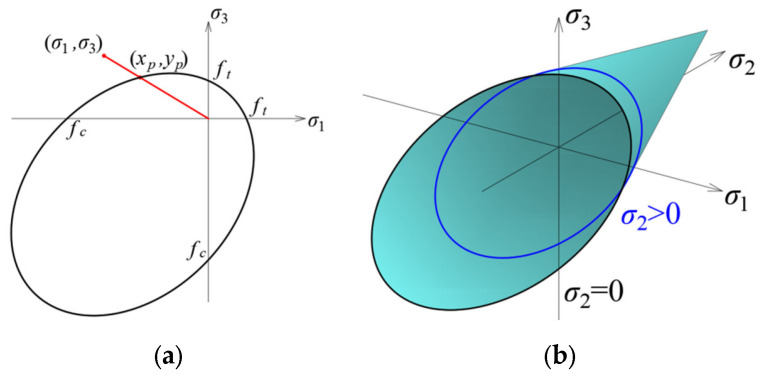
Envelope of the Drucker–Prager criterion in the plane of principal stresses: (**a**) in a two-dimensional stress state; (**b**) in a three-dimensional stress state.

The implementation of the Drucker–Prager criterion was almost identical to that described above for the Coulomb–Mohr criterion. Also, in this case, Formula (2) was applied for the 2D stress state, but the coordinates of the intersection of the stress line and the envelope are calculated differently since the shape of the envelope in this coordinate system is an ellipse (Figure 9a):

(6) $$x_{p}=\frac{-2\eta \left(\eta +\kappa -\eta \kappa \pm \left(\eta +1\right)\sqrt{\kappa^{2}+\kappa +1}-1\right)}{3\eta^{2}\kappa +4\eta \kappa^{2}-2\eta \kappa +4\eta +3\kappa }f_{c},y_{p}=-\kappa x_{p},$$

Also, in this case, the envelope differ if the middle principal stresses are different from 0 (Figure 9b). In the case of the Drucker–Prager criterion, a formula analogous to (4) are used:

(7) $$\mu =\frac{1}{f_{c}}\left(\frac{\eta -1}{2}\left(\sigma_{1}+\sigma_{2}+\sigma_{3}\right)+\frac{\eta +1}{2}\sqrt{\frac{{\left(\sigma_{1}-\sigma_{2}\right)}^{2}+{\left(\sigma_{2}-\sigma_{3}\right)}^{2}+{\left(\sigma_{3}-\sigma_{1}\right)}^{2}}{2}}\right),$$

For use in the Abaqus^®^ system, only the method described by the above formula was programmed for the Drucker–Prager criterion.

### 2.5. Ottosen–Podgórski Criterion

**Figure 10:**
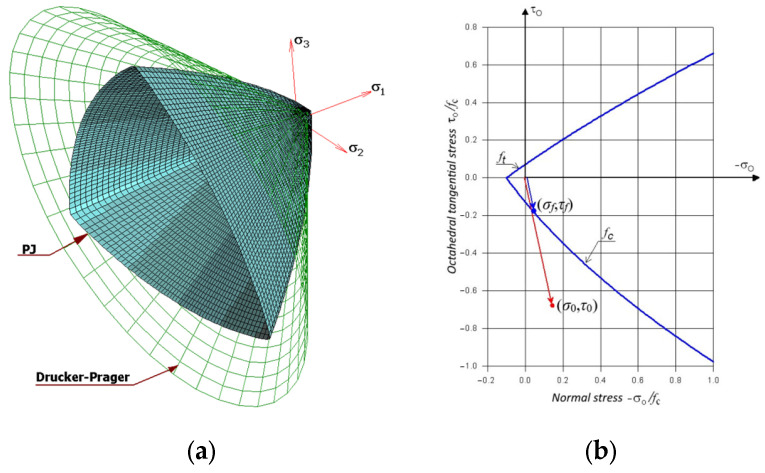
Envelope of the Ottosen–Podgórski (PJ) criterion: (**a**) in a three-dimensional state of stress; (**b**) in the *σ*_0_-*τ*_0_ plane.

The Ottosen–Podgórski criterion [20] was proposed in a form containing three stress tensor invariants:

(8) $$\sigma_{0}-C_{0}+C_{1}P\left(J\right)\tau_{0}+C_{2}\tau_{0}^{2}=0,$$

where *P*(*J*) is a function describing the cross-section of the criterion in a deviatoric plane where *σ*_0_ = const.:

(9) $$P\left(J\right)=\cos\left(\frac{1}{3}\mathrm{acos}\xi J-\varphi \right),$$

where ξ, φ, *C*_0_, *C*_1_, *C*_2_—parameters obtained from material constants. More information about the discussed criterion can be found in publications [15,20,21] and Appendix A. The shape of the criterion envelope in the three-dimensional stress state is shown in Figure 10a. The material effort itself can be determined from the following ratio:

(10) $$\mu =\frac{\sqrt{\sigma_{0}^{2}+\tau_{0}^{2}}}{\sqrt{\sigma_{f}^{2}+\tau_{f}^{2}}},$$

where

*σ*_0_, *τ*_0_—normal and tangential stresses occurring at a given point;

*σ_f_*, *τ_f_*—normal and tangential stresses proportional to the above. This is a point located on the criterion surface. The above stress values are also shown in Figure 10b.

### 2.6. Hoek–Brown Criterion

The Hoek–Brown criterion was originally proposed in 1980 [22] in the form:

(11) $$\sigma_{1}=\sigma_{3}+\sqrt{A\sigma_{3}+B^{2}},$$

where *A* and *B* are material constants:

(12) $$A=\frac{f_{c}^{2}-f_{t}^{2}}{f_{t}},B=f_{c},$$

**Figure 11:**
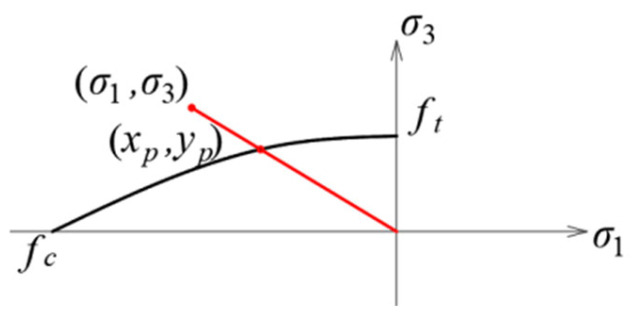
The envelope of the Hoek–Brown criterion in the plane of principal stresses in a plane stress state.

As for the Coulomb–Mohr and Drucker–Prager criteria, the Hoek–Brown criterion is described in a 2D stress state in the plane of principal stresses [23,24]. The shape of the criterion envelope in this plane is shown in Figure 11. Here, too, the material effort could be determined using Formula (2), but the point of intersection of the stress line with the envelope could be determined from the formulas obtained after transforming Formula (11):

(13) $$x_{p}=\frac{f_{c}}{2{\left(\kappa -1\right)}^{2}}\left(\eta \kappa \pm \sqrt{\eta^{2}\kappa^{2}+4{\left(\kappa -1\right)}^{2}}\right),y_{p}=-\kappa x_{p},$$

Unfortunately, due to insufficient information in the literature, the analysis for the three-dimensional stress state was not performed, so in simulations with three stress directions, the above failure criterion was used for the 2D stress state.

### 2.7. Comparison of All Criteria

**Figure 12:**
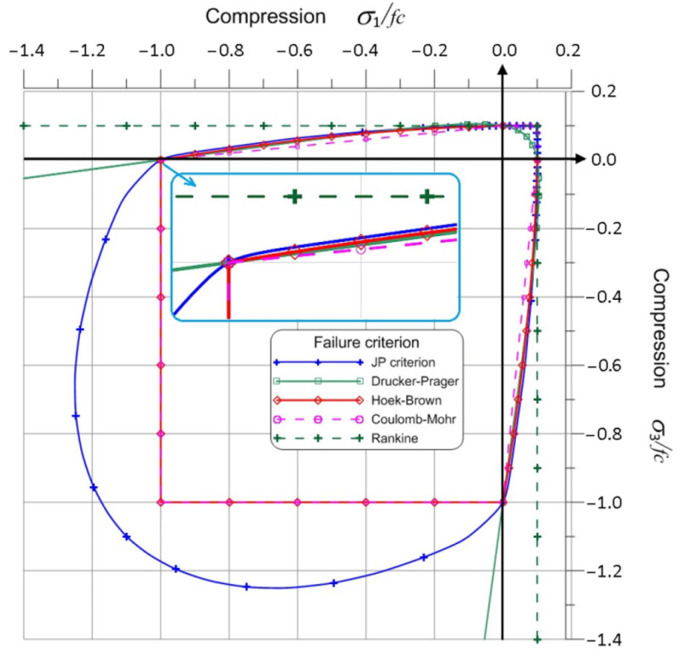
Envelopes of all failure criteria in the plane of principal stresses.

Figure 12 summarizes all the analyzed material failure criteria in the plane of principal stresses for the ratio *f_c_*/*f_t_* = 10.

As can be observed, in the compression–tension quadrant, the criteria are slightly different from each other. The Rankine criterion is the upper limit of the envelope, and the Coulomb–Mohr criterion is the lower limit. Hence, the Rankine criterion should give the lowest value of material effort and the Coulomb–Mohr criterion the highest. In the tension–tension quadrant, only the Drucker–Prager criterion differs from the others. For this criterion, a much higher value of the material effort should be obtained than for the other criteria.

In order to verify the correctness of the above-described formulas for all criteria, it was decided to determine the material effort values for three real exemplary stress tensors:

(14) $$\sigma_{A}=\left[\begin{array}{ccc} 3 & 0 & 0\\ 0 & -100 & 0\\ 0 & 0 & 0 \end{array}\right]MPa,\sigma_{B}=\left[\begin{array}{ccc} 2.28 & 0.19 & 0\\ 0.19 & 1.72 & 0\\ 0 & 0 & 0 \end{array}\right]MPa,\sigma_{B}=\left[\begin{array}{ccc} 2.02 & -0.44 & 0\\ -0.44 & 3.25 & 0\\ 0 & 0 & 0.55 \end{array}\right]MPa,$$

The following material parameters were selected: *f_c_* = 92.56 MPa, *f_t_* = 3.11 MPa. These are the material parameters of the “Brenna” sandstone, which was the subject of laboratory tests in the authors’ previous works. The first stress tensor is a simple example of principal stresses in the tension–compression quadrant. The remaining two are actual stress values read randomly from simulations performed for the purposes of this publication, respectively, for the 2D and the axisymmetric tasks.

Table 1 lists the above-described values of the material effort. In the case of a plane stress state, both ways of determining the coefficient (as for a plane stress state and for a three-dimensional stress state) were the same for the Coulomb–Mohr and Drucker–Prager criteria. However, for tensor C, i.e., for a three-dimensional task, the value of the coefficient determined in the same way as for a flat task was obviously incorrect, but the difference was not very significant. In each case, the material effort obtained the minimum value for the Rankine criterion and the maximum value for the Coulomb–Mohr criterion. For tensors B and C (biaxial stretching), the coefficient values obtained for the Drucker–Prager criterion differed significantly from the other criteria. All the assumptions deduced at the beginning of this chapter are, therefore, true.

**Figure 13:**
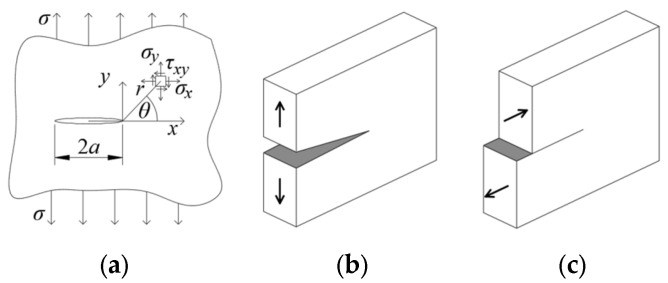
The crack analyzed by Griffith (**a**), and crack loading schemes Mode I (**b**), Mode II (**c**).

It may also be interesting to analyze the material effort and, thus, the crack propagation angle based on theoretical data. A Griffith’s crack was used, i.e., a crack placed on an infinite disk loaded with a uniform load. A diagram of this theoretical task is shown in Figure 13a. This crack can be loaded perpendicularly—in Mode I of loading (Figure 13b) or longitudinally—in Mode II (Figure 13c).

The stresses around the crack tip in both modes were solved by Westergaard [16] and are described by the following formulas:

(15) $$\sigma_{x.I}\left(r,\theta \right)=\frac{K_{I}}{\sqrt{2\pi r}}\cos\left(\frac{\theta }{2}\right)\left(1-\sin\left(\frac{\theta }{2}\right)\sin\left(\frac{3\theta }{2}\right)\right),\;\sigma_{y.I}\left(r,\theta \right)=\frac{K_{I}}{\sqrt{2\pi r}}\cos\left(\frac{\theta }{2}\right)\left(1+\sin\left(\frac{\theta }{2}\right)\sin\left(\frac{3\theta }{2}\right)\right),\;\tau_{xy.I}\left(r,\theta \right)=\frac{K_{I}}{\sqrt{2\pi r}}\cos\left(\frac{\theta }{2}\right)\sin\left(\frac{\theta }{2}\right)\cos\left(\frac{3\theta }{2}\right),\;\sigma_{x.II}\left(r,\theta \right)=-\frac{K_{II}}{\sqrt{2\pi r}}\sin\left(\frac{\theta }{2}\right)\left(2+\cos\left(\frac{\theta }{2}\right)\cos\left(\frac{3\theta }{2}\right)\right),\;\sigma_{y.II}\left(r,\theta \right)=\frac{K_{II}}{\sqrt{2\pi r}}\sin\left(\frac{\theta }{2}\right)\cos\left(\frac{\theta }{2}\right)\cos\left(\frac{3\theta }{2}\right),\;\tau_{xy.II}\left(r,\theta \right)=\frac{K_{II}}{\sqrt{2\pi r}}\cos\left(\frac{\theta }{2}\right)\left(1-\sin\left(\frac{\theta }{2}\right)\sin\left(\frac{3\theta }{2}\right)\right).$$

where *K*_I_ and *K*_II_ are the critical stress intensity factors for the material, taken as 1, *r*—distance from the crack tip, taken as 1, *θ*—angle around the crack tip.

**Figure 14:**
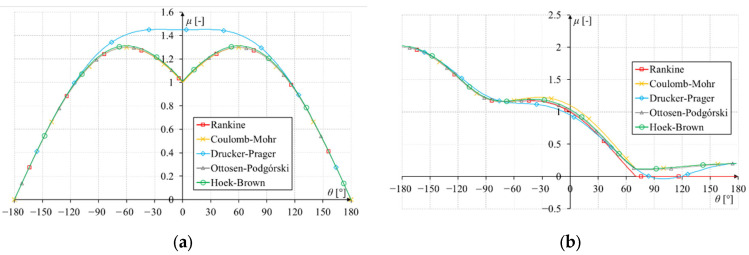
Plots of the material effort for stresses around the tip of the Griffith’s crack: (**a**) for Mode I; (**b**) for Mode II.

The principal stresses and octahedral stresses were determined for the above functions, and on their basis, formulas for the material effort functions were determined for all the discussed criteria. The ratio *f_c_*/*f_t_* was also assumed to be 10. The plots of these functions for both loading modes are shown in Figure 14. It turns out that for Mode I, the local minimum was achieved for the angle *θ* = 0°. This is understandable considering that Mode I stress diagrams are symmetrical. It was different for Mode II. The angle for which the local minimum of the function was achieved for each criterion differed slightly: Rankine −70.529°, Coulomb–Mohr −70.534°, Ottosen–Podgórski −70.815°, Hoek–Brown −70.584°. The Drucker–Prager criterion did not reach a local minimum in this area due to its characteristics (the intersection of the cone with the plane stress state gives an ellipse as in Figure 9a) in the tension–tension quadrant. The D-P criterion differed significantly from the other criteria, as shown in Figure 12. Moreover, this angle changes slightly depending on the *f_c_*/*f_t_* ratio.

## 3. Results

### 3.1. Three-Point Bending Test of a Notched Beam

**Figure 15:**
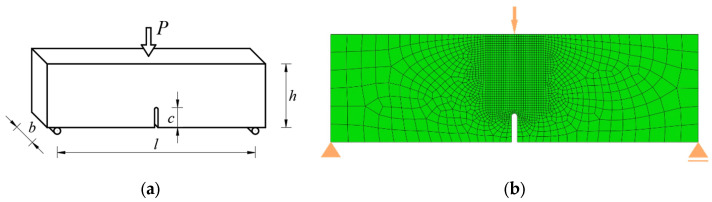
Three-point bending test of a beam with a notch: (**a**) scheme of the task; (**b**) finite element mesh.

A three-point bending test of a notched beam was the subject of the authors’ research. The present work contains a description of laboratory tests of material parameters and the results of the described test. The test scheme is shown in Figure 15a, and the finite element mesh in Figure 15b. The selected material was sandstone with the following material parameters: Young’s modulus *E* = 13.72 GPa, Poisson’s ratio *ν* = 0.1482, compressive strength *f_c_* = 92.56 MPa, tensile strength *f_t_* = 3.110 MPa, critical strain energy release rate in Mode I *G*_I0_ = 0.04794 N/mm. The dimensions of the sample are length *l* = 320 mm, width *b* = 90.2 mm, height *h* = 93.7 mm, and notch depth *c* = 25 mm.

**Figure 16:**
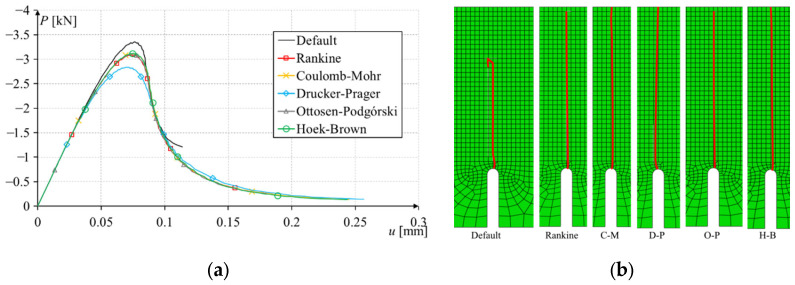
Simulation results of three-point bending of a beam with a notch: (**a**) force–displacement plot; (**b**) crack paths.

In computer simulations, the load was controlled by a vertical displacement at a node at the center of the top edge. The sample was modeled in a plane stress state, with supports modeled in the lower left and right corners. The mesh in the crack area was modeled as uniform and rectangular. The size of finite elements ranges from 1 to 15 mm. The obtained force–displacement plots and crack paths are shown in Figure 16.

The nature of the damage was the same for all criteria. The maximum force was similar, and only for the default Abaqus^®^ criterion was overestimated. The Drucker–Prager criterion gave an underestimated result since this was a 2D task, and the stresses around the crack tip were mainly tensile stresses. The crack path for the default Abaqus^®^ method was only initially consistent with the trajectory predicted by the analytical solutions. After crossing about 2/3 of the path, it unexpectedly turned (Figure 16b), which is probably related to the highly simplified method of estimating the stress field around the crack tip. This change in trajectory led to an increase in the value of the force required for further crack propagation (Figure 16a) and the inability to drive the crack to the top edge of the model. All programmed criteria generated very correct crack paths, maintaining symmetry and brought to the edge of the model. This crack path was consistent with the results obtained in the laboratory, as described in the publication [8]. Unfortunately, the maximum force obtained was far from the laboratory test result due to the heterogeneity of the sandstone, as mentioned in a previous publication [25].

### 3.2. Pull-Out Test of a Self-Undercut Anchor

**Figure 17:**
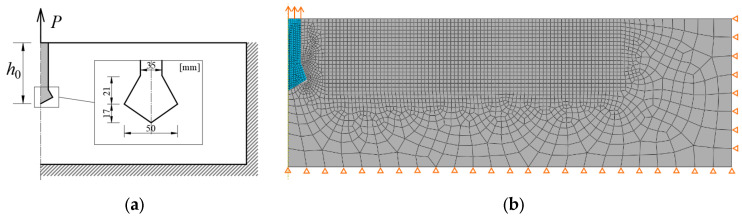
Pull-out test of a self-undercutting anchor: (**a**) scheme of the task; (**b**) finite element mesh.

The self-undercutting anchor pull-out test has also been described in previous publications [8]. This is an axisymmetric task. The task diagram and the finite element mesh are presented in Figure 17. The material used was the same as for the study above, and the anchor length was *h*_0_ = 10 cm.

**Figure 18:**
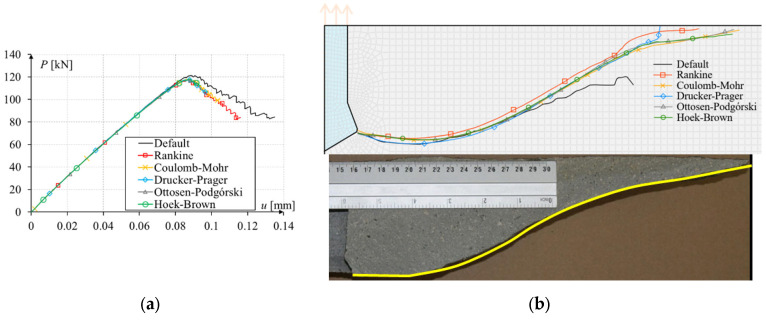
Simulation results of the pull-out test of a self-undercutting anchor: (**a**) force–displacement plot; (**b**) crack paths after simulation compared with a rock sample pulled out with a self-undercutting anchor.

In the simulations, the load was controlled by vertical displacement. In situ tests were performed on a mass of rock, so the model was chosen to be large enough so the bottom and right edges of the model would not have a significant impact on the fracture. Boundary conditions were created on the lower and right edges of the model, and on the left edge, there was an axis of rotation. The size of the finite element mesh ranged from 2 mm near the expected beginning of the crack to 25 mm. The results of computer simulations, such as the plot of the force–displacement relation and the obtained crack paths, are shown in Figure 18.

The relationship between the pull-out force and the vertical displacement was again very similar for all criteria. Again, the default method gave slightly overestimated results. As for the crack line, again, the default method gave an incorrect result. However, the programmed criteria led the line correctly and very similarly to each other. As proven in previous publications, the crack line and the destructive force for the authors’ method coincided with the results of in situ tests [8] (the pull-out force obtained on in situ test was 123 kN).

### 3.3. Pull-Out Test on Rod with Thread

**Figure 19:**
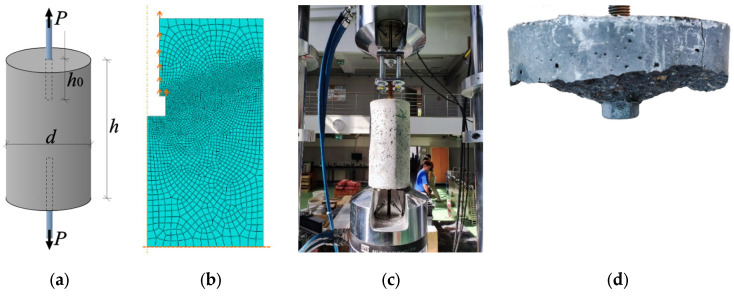
Pull-out test of a rod embedded in concrete: (**a**) scheme of the task; (**b**) finite element mesh; (**c**) laboratory test; (**d**) exemplary sample after laboratory test.

Another test, also described in the authors’ previous publication, is a pull-out test of a rod embedded in a concrete cylinder [25]. This study was different from the above. Here, there was contact between the rod thread and the concrete, whereas above, the load was carried out only by the pressure of the anchor undercut on the rock. This time, the material parameters were Young’s modulus *E* = 38.000 GPa, Poisson’s ratio *ν* = 0.2, compressive strength *f_c_* = 44.013 MPa, tensile strength *f_t_* = 3.300 MPa, critical fracture energy in Mode I *G*_I0_ = 0.015 N/mm. The task scheme is shown in Figure 19a. The sample dimensions were width *d* = 150 mm, height *h* = 300 mm, and screw mounting depth *h*_0_ = 5 cm. A nut was attached to the end of the screw. The load was controlled by vertical displacement through the contact of the thread with the concrete and the pressure of the nut on the concrete. The test was modeled as a 1/4 cross-section of a cylinder, as an axially symmetrical test. On the left and bottom edges, there were axes of symmetry, with the left one being the axis of rotation. The finite element mesh is shown in Figure 19b. The size of finite elements was from 2 to 5 mm.

**Figure 20:**
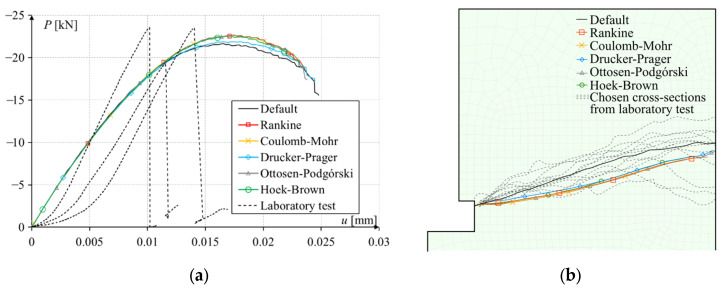
Simulation results of the pull-out test of a rod embedded in concrete: (**a**) force–displacement plot; (**b**) crack paths.

The results of computer simulations are shown in Figure 20, which are a force–displacement relationship plot and the crack paths. Again, the results of the maximum force and the nature of damage for all criteria were similar, and the fracture lines for all programmed criteria were almost identical, while the result for the simulation with the default method differed from the others. Unlike the previous two simulations, the default method brought the crack to the edge of the model. The crack path was similar to the line obtained in the laboratory tests (compare Figure 19a and Figure 20b), and the maximum force was also consistent (Figure 20a). Unfortunately, there was a large difference in the nature of failure (force–displacement relationship). This problem was described in the previous publication [25] and was not caused directly by the programmed criteria. The most likely reason for this was incorrectly assumed fracture energy. For a lower value of the fracture energy in the simulations, the value of the force needed to initiate the fracture decreased. Boundary conditions also turned out to be a problem. Another reason for some inconsistencies was the inability to properly model the connection between the thread and the concrete. The different methods of modeling the load transfer to the concrete did not significantly affect the results, but only in the case of simulations with an elastic rod did the shape of the force–displacement relationship curve turn out to be similar to that obtained in tests.

## 4. Discussion

This paper presents the results of numerical simulations of several laboratory tests performed on specimens made of rock-like materials. These included tests of three-point bending of a notched beam cut from sandstone, the pull-out of a self-undercutting anchor fixed in sandstone, and a pull-out test of a bar embedded in a cylindrical concrete specimen. The simulations were performed using the commercial Simulia Abaqus^®^ system, in which the UDMGINI subroutine responsible for determining crack propagation directions was modified by the authors. The reason for undertaking this modification was the observed inconsistency in the fracture path obtained by the standard procedure available in the system, which, in addition to the faulty shape, also resulted in the inability to obtain the extent of the pulled-out rock fragment in the pull-out test.

Analysis of the standard procedure identified two reasons for these inconsistencies. The first was a very simple method of predicting the direction of fracture propagation, which equates this orientation with the direction of principal stress, which only works in the first fracture mode. The second reason was an insufficiently accurate method for determining the principal stress directions near the crack tip, which was based on stress analysis in one finite element adjacent to the tip. The programmed UDMGINI procedure analyzes the stresses read at the integration points of the elements lying in a larger area than the standard procedure, so the process of determining the stress field gave a better estimate of the principal directions. Determination of the direction of crack propagation was based on the search for the minimum gradient of the material effort field, which depends on the adopted material failure criterion. Such a method proposed in the work [15] gives a very good estimate of the propagation direction and is easier to implement than methods based on stress intensity factors, as shown in the work [8].

In the current work, several different failure conditions commonly used in rock and concrete mechanics were used in the simulations: Rankine, Coulomb–Mohr, Drucker–Prager, Ottosen–Podgórski, and Hoek–Brown criteria. All programmed criteria produce significantly better results than the default maximum principal stress criterion available in the Abaqus^®^ system. The advantage over the default method is a smoother crack path that is less dependent on the finite element mesh and brings the fracture to the end of the simulation, which coincides with the results of laboratory and in situ tests. In the pull-out test of the self-undercutting anchor, the maximum force result was much closer to the actual result, as shown in previous publications.

All programmed criteria give very similar results, which allows us to assume that they were implemented correctly. For the needs of this publication, the method of selecting integration points around the crack tip and the polynomial fitting to the stresses at these points were also refined, which allowed for better results than in simulations using the authors’ method described in previous publications.

The main goal of the work was achieved. The authors’ method enables easy implementation of any stress-based material failure criterion in fracture simulations using the X-FEM method in Abaqus^®^. Converting the subroutine code of the previously programmed Rankine criterion to the remaining criteria took the authors several minutes of coding, plus some testing and the debugging process. This means that this method will allow researchers who are developing new material failure criteria to quickly check their effectiveness.

The authors plan to implement other types of criteria in the next stage of the work. Since the criteria discussed here require a well-defined stress field around the crack tip, which can often be difficult, they plan to investigate criteria not based directly on stress.

The question of the incompatibility of simulations performed for an isotropic material model with experiments using concrete specimens, which were definitely a significantly inhomogeneous material due to their dimensions and aggregate sizes, is the subject of our further research. The results of the experiments and simulations will be presented in our next papers. Such simulations using our own software have already been published in earlier papers [15], but their implementation in a commercial system poses some difficulties, which we will try to overcome.

## 5. Conclusions

The numerical simulations presented in this paper, performed with the commercial Abaqus^®^ system, showed the inadequacies of the simple method of determining the direction of crack propagation modeled by the X-FEM method. This was particularly evident in areas with complex stresses, where I-Mode cannot be used in fracture analysis. In areas with a dominant tensile principal stress, the standard procedure gave satisfactory results. The UDMGINI procedure programmed by the authors, which is responsible for determining the direction of crack propagation, performed much better, allowing a more accurate determination of the crack path, as well as the maximum value of the force destroying the specimen. Simulation of the pull-out test for a sandstone fragment using a self-undercutting anchor clearly showed the advantage of the improved procedure, which takes into account the criterion of the minimum gradient of the material effort field around the crack tip.

Simulations of highly inhomogeneous materials using the isotropic material model available in the standard X-FEM implementation cannot show the exact geometry of the crack path. The apparent effect of scale, which can be characterized by the ratio of the maximum inhomogeneity size to the specimen size (in concrete, it is the maximum aggregate granularity to the minimum specimen size), should be taken into account when creating an FEM model. As it currently stands, the proposed procedure allows for the analysis of cracking in 2D models (including axisymmetric models). It would be desirable to extend it with the possibility of analyzing 3D phenomena.

The problem, as it seems at present, is also the analysis of bifurcation cracks, which is related to the implementation of the X-FEM method available in Abaqus^®^, which requires the use of another specialized system or own software.

## Figures and Tables

**Table 1:** Material effort for three exemplary stress tensors for all criteria.

Criterion	Material Effort for Tensor:		
	A	B	C
Rankine	0.965	0.752	1.092
Coulomb–Mohr (2D approach—linear function)	2.045	0.752	1.092
Coulomb–Mohr (3D approach)	2.045	0.752	1.086
Drucker–Prager (2D approach—ellipse)	1.801	0.968	1.137
Drucker–Prager (3D approach)	1.801	0.968	1.305
Ottosen–Podgórski	1.665	0.752	1.092
Hoek–Brown	1.695	0.752	1.093

## Data Availability

Data are available in a publicly accessible repository.

## Appendix A

The Ottosen–Podgórski criterion was proposed in a form containing three stress tensor invariants:

(A1) $$\sigma_{0}-C_{0}+C_{1}P\left(J\right)\tau_{0}+C_{2}\tau_{0}^{2}=0,$$

where

$P\left(J\right)=\cos\left(\frac{1}{3}\mathrm{acos}\xi J-\varphi \right)$ is a function describing the cross-section of the criterion in the deviatoric plane where $\sigma_{0}$= const.;

$\sigma_{0}=\frac{1}{3}I_{1}$—mean stress;

$\tau_{0}=\sqrt{\frac{2}{3}J_{2}}$—octahedral shear stress;

$I_{1}$—first invariant of the stress tensor;

$J_{2},\;J_{3}$—second and third invariant of the stress deviator;

$J=\frac{3\sqrt{3}J_{3}}{2J_{2}^{3/2}}$—alternative invariant of the stress deviator;

$\xi ,\;\varphi ,\;C_{0},\;C_{1},\;C_{2}$—material constants.

Classical failure criteria, like Huber–Mises, Tresca, Drucker–Prager, Coulomb–Mohr, as well as some more recent ones proposed by Lade, Matsuoka, and Ottosen, are special cases of the general form (A1) of this criterion.

Material constants can be evaluated on the basis of some simple material test results like:

*f_c_*—failure stress in uniaxial compression;

*f_t_*—failure stress in uniaxial tension;

*f_cc_*—failure stress in biaxial compression at $\sigma_{1}/\sigma_{2}=1$;

*f*_0*c*_—failure stress in biaxial compression at $\sigma_{1}/\sigma_{2}=2/1$;

*f_v_*—failure stress in triaxial tension at $\sigma_{1}/\sigma_{2}/\sigma_{3}=1/1/1$;

For concrete or rock-like materials, some simplification can be taken on the basis of the Rankine–Haythornthwaite “tension cutoff” hypothesis: $f_{v}=f_{t}$.

Values of the material constants *C*_0_, *C*_1_, *C*_2_ can be calculated from following equations:

(A2) $$C_{0}=f_{t},\;\;\;C_{1}=\frac{\sqrt{2}}{P_{0}\;}\left(1-\frac{3}{2\;}\;\frac{f_{t}/f_{cc}}{f_{cc}/f_{t}-1}\right),\;\;\;C_{2}=\frac{9}{2}\;\frac{f_{t}/f_{cc}}{f_{cc}-f_{t}}$$

where $P_{0}=\cos\left(\frac{1}{3}\arccos\xi -\varphi \right)$.

The values of the parameters *ξ* and φ can be calculated from the following equations:

(A3) $$\xi_{0}=acos\left[\frac{\theta }{2}\left(1+\frac{1}{\lambda }\right)\right],\;\;\;\varphi =\frac{\pi }{6}-atan\left[\frac{\theta \left(1-\lambda \right)}{2\lambda \;sin\xi_{0}}\right],\;\;\;\xi =sin\left(3\xi_{0}\right),$$

where

$$\lambda =\frac{f_{cc}}{f_{t}}\frac{\frac{1}{3}+\frac{f_{t}}{f_{c}}-\frac{f_{t}f_{c}}{\left(1-f_{t}/f_{cc}\right)f_{cc}^{\;2}\;}}{1+\frac{2f_{cc}}{3f_{t}}-\frac{1}{1-f_{t}/f_{cc}}},\;\theta =\frac{\sqrt{3}f_{0c}}{2f_{t}}\frac{\frac{1}{3}+\frac{f_{t}}{f_{c}}-\frac{f_{t}f_{c}}{\left(1-f_{t}/f_{cc}\right)f_{cc}^{\;2}\;}}{1+\frac{f_{0c}}{2f_{t}}-\frac{3f_{0c}^{\;2}}{4\left(1-f_{t}/f_{cc}\right)f_{cc}^{\;2}}}\;.$$
